# Adaptive tuning functions arise from visual observation of past movement

**DOI:** 10.1038/srep28416

**Published:** 2016-06-24

**Authors:** Ian S. Howard, David W. Franklin

**Affiliations:** 1 Centre for Robotics and Neural Systems, School of Computing, Electronics and Mathematics, University of Plymouth, Plymouth, United Kingdom; 2 Neuromuscular Diagnostics, Department of Sport and Health Science, Technical University of Munich, Munich, Germany

## Abstract

Visual observation of movement plays a key role in action. For example, tennis players have little time to react to the ball, but still need to prepare the appropriate stroke. Therefore, it might be useful to use visual information about the ball trajectory to recall a specific motor memory. Past visual observation of movement (as well as passive and active arm movement) affects the learning and recall of motor memories. Moreover, when passive or active, these past contextual movements exhibit generalization (or tuning) across movement directions. Here we extend this work, examining whether visual motion also exhibits similar generalization across movement directions and whether such generalization functions can explain patterns of interference. Both the adaptation movement and contextual movement exhibited generalization beyond the training direction, with the visual contextual motion exhibiting much broader tuning. A second experiment demonstrated that this pattern was consistent with the results of an interference experiment where opposing force fields were associated with two separate visual movements. Overall, our study shows that visual contextual motion exhibits much broader (and shallower) tuning functions than previously seen for either passive or active movements, demonstrating that the tuning characteristics of past motion are highly dependent on their sensory modality.

Humans adapt to changes in dynamics by developing an internal model or motor memory which can predictively compensate for the new dynamics^1^,^2^,^3^. This is often investigated experimentally by having participants making reaching movements while a robotic device applies viscous force fields to their hand^1^,^4^,^5^. Such learning is not selective to the specific trained movement, but generalizes to both different movements^2^ and different spatial regions^1^,^6^,^7^,^8^. This predictive generalization of the learned dynamics decays as a function of angle^9^,^10^ and distance^4^,^11^,^12^ away from the trained movement direction.

When participants perform movements with consistent environmental dynamics (e.g. a curl force field), rapid learning of the motor memory occurs, resulting in a correspondingly rapid reduction in movement error. However, if the movements are performed in opposing dynamics (e.g. opposing direction curl-fields), which alternate or randomly change direction from one movement to the next, interference occurs such that neither curl-field is learned^4^,^5^,^13^,^14^,^15^,^16^. Instead, the motor memory attempts to learn the mean of the force fields that have been applied on the recent trials^17^, which in the case of balanced alternating curl fields is zero. This interference can be reduced by associating appropriate contextual cues with the curl-field direction, providing the motor system with extra information and thereby enabling the partitioning of the tasks into separate motor memories^16^,^18^,^19^,^20^,^21^,^22^,^23^,^24^. Specifically, many of these studies have shown that the strongest reduction in interference occurs when the state information of the limb is either visually or physically separated during the adaptation movement^16^,^20^,^21^,^25^. However, we have recently shown that even when the limb state is matched during the adaptation movement, interference can be avoided if different past movements (lead-in)^26^ or different future movements (follow-through)^27^ are associated with each of the curl-field directions. In these studies the adaptation movement in a curl field was either preceded or followed by a contextual movement. The preceding contextual movement was equally effective whether it was generated actively, passively, or was a purely visual movement^26^. These studies suggested that the specific predictive motor memory that is active for each movement depends not only on the limb states during the adaptive movement, but also on limb states either preceding or following the adaptive movement. Interestingly, this effect disappeared as the time between movements increased such that it was effectively absent when the movements were separated by more than about half a second^26^.

It has been previously shown that motor memories generalize away from the adaptive movement as the direction is varied^9^,^10^. In a recent study we demonstrated that passive contextual movement shows similar generalization responses. It also produces a tuning function as the contextual movement direction is varied, even when learning a single force field^28^. In fact we found similar widths of the tuning functions for both the contextual and adaptive movement, although the contextual movement tuning was shallow and did not decay completely as the movement direction rotated beyond ninety degrees. This finding was further supported by a similar study in which the preceding contextual movement was a combination of active and visual motion^24^ in contrast to purely passive movements^28^. Moreover, both of these studies examined the pattern of generalization after participants learned to uniquely associate two separate contextual movements with two opposing curl force fields in an interference task. It was suggested that the pattern of interference and generalization in this task could be predicted by the contextual and adaptive tuning functions measured in the single force field experiment^28^.

Although it has been shown that preceding contextual motion allows independent learning of opposing force fields whether it is passive, active or visual in nature^26^, it is well know that vision and proprioception can have different effects during movement^29^. Many studies have demonstrated the key role of proprioception in driving adaptation^30^,^31^,^32^. However, recent work has also highlighted a distinct role for visual feedback in dynamic adaptation^29^,^33^,^34^. Moreover, the contribution of visual and proprioceptive signals can be flexibly weighted depending on properties such as noise or uncertainty^35^,^36^,^37^. As the accuracy of both vision and proprioception vary with each other and throughout the workspace^38^,^39^, the sensitivity of these contextual signals may vary across sensory modalities. To investigate this question, here we characterized the angular tuning responses for past visual contextual movements in a set of experiments looking at adaptation to both a single force field and in an interference study. We first estimate the directional tuning response curves of the contextual and adaptation movement in a single curl-field learning task. Second, we use interference experiments to investigate the tuning response obtained when we apply opposing curl fields, each associated with two different contextual motion cues. Our previous work has shown that the tuning of the contextual movement plays its biggest role in motor memory formation when past contextual information is crucial to the task, such as in an interference study. Therefore, here we specifically examine whether the tuning function for the contextual movement or adaptation movement can be used to predict the pattern of generalization found in the interference experiment. In order for us to be able to make direct comparisons with prior studies, here we use a similar paradigm to our previous study^28^. The main distinctions are that the contextual movements are now visual in nature (rather than passive physical movements) and modeling of the angular tuning functions are performed using von Mises functions (rather than using Gaussian functions).

## Results

### Experiment 1: Generalization of a single motor memory

**Figure 1:**
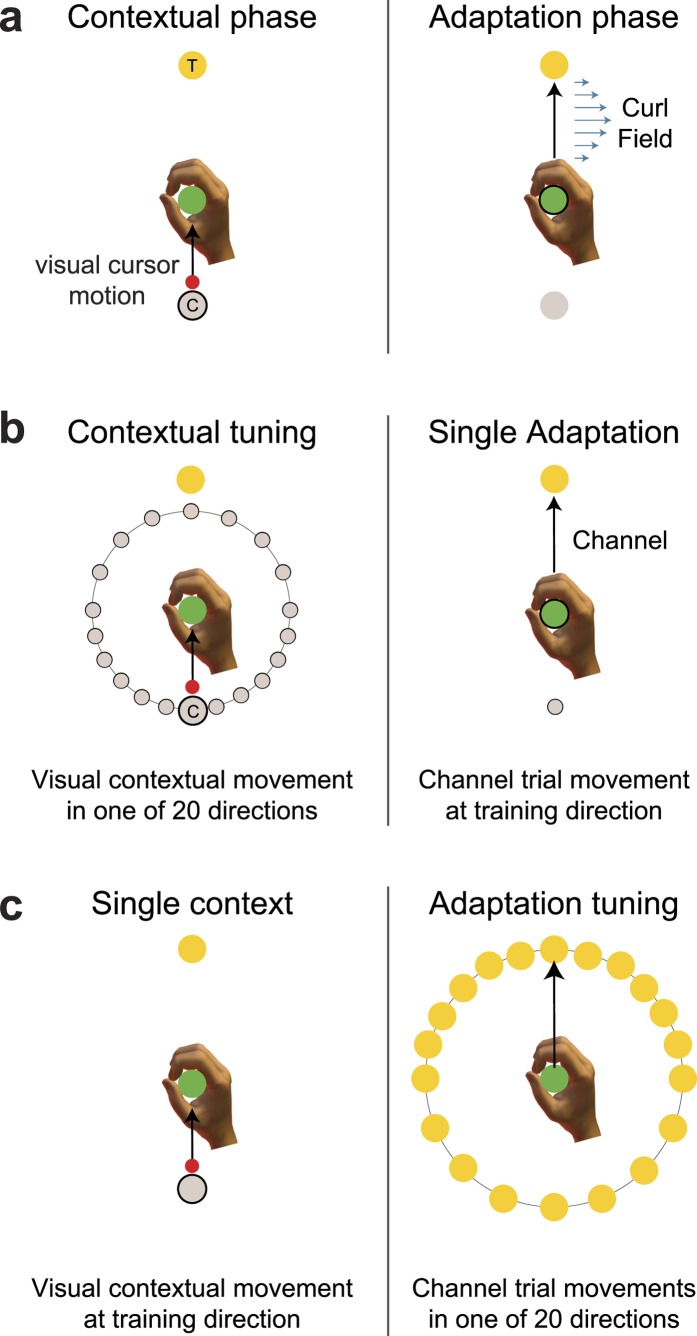
Single field tuning experimental paradigm. (**a**) Starting with the participant’s hand at the central location (green circle), the contextual phase consisted of visual observation of a cursor moving from its start location (grey shaded circle) to the central location. Immediately afterwards in the adaptation phase, the participant was required make an active movement to a final target location (yellow circle), during which they were subject to a curl force field. (**b**) To examine contextual movement direction generalization, the visual cursor start position was varied while the adaptation movement was made to a single target in a mechanical channel to measure the predictive force. (**c**) To examine adaptation movement direction generalization, the visual cursor start position was fixed to the single location used during training and the adaptation movement direction was varied and performed in a mechanical channel to measure the predictive force.

**Figure 2:**
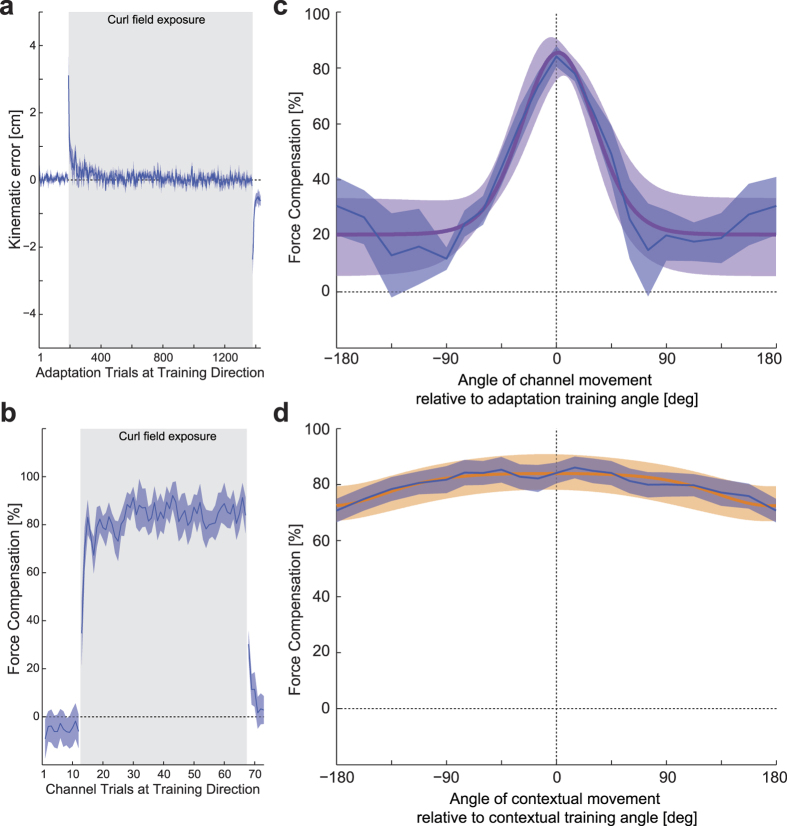
Single field tuning experimental results. (**a**) Mean kinematic error and SE per block across participants (solid line and shaded region respectively) for the adaptation movements. The light blue shading indicates when the curl force field is applied. (**b**) Percentage mean and SE of predictive force compensation measured using a channel towards the training target. (**c**) Adaptation movement tuning curve expressed as mean and SE percentage compensation (blue). The mean and s.d. of a von Mises fitted curve are plotted as a purple line and shaded purple region respectively. (**d**) Contextual movement tuning curve expressed as mean and SE percentage compensation (blue). The mean and s.d. of a von Mises fitted curve are plotted as a orange line and shaded orange region respectively.

The first experiment examined the directional tuning of both contextual visual motion and the active adaptive movements using a single curl field-learning task. In this experiment the past visual motion context is not required in order to learn the compensation (Fig. 1). Each trial consisted of a two-part movement. While the participant’s hand remained at the central location, participants experienced a 10 cm visual contextual movement of the cursor from a starting position to the central location. Immediately afterwards the participants were required to make a 12 cm active adaptation movement from this central location to a final target located straight ahead. This active movement could be performed in a null field, a mechanical channel, or a curl force field (Fig. 1a right). During the initial pre-exposure phase, movements were essentially straight (Fig. 2a). After field exposure onset, MPE increased dramatically with a rapid adaptation back towards null field MPE levels. After the curl-field was removed during the post-exposure phase, strong after-effects were seen which quickly washed-out. Predictive force compensation (Fig. 2b) also indicated rapid learning during field exposure, which rose to over 80% by the end of exposure and quickly decayed after field removal. The dwell time between the prior visual contextual movements and the adaptation movements was 78.7 ± 9.3 ms (mean ± SE).

After participants had learned to compensate the curl field, channel trials were used to examine generalization to changes in contextual and adaptation movement directions. To examine the generalization effect of contextual movement direction, the final target location remained fixed at the 0° target and the location of the visual contextual motion starting point was varied (Fig. 1b). To examine the generalization effect of adaptation movement direction, the visual contextual motion training direction remained fixed at the training location while the target locations varied (Fig. 1c). The resulting tuning curves for both adaptation and contextual movements, plotted in terms of mean and SE percentage compensation across subjects, are shown in Fig. 2c (blue curve) and 2d (blue curve) respectively. The form of the adaptation curve (Fig. 2c) is consistent with observation made by other researchers, exhibiting a reduction to around 20% compensation at ±90° angle^9^, which may represent a non-specific or global portion of the compensation^24^. Outside this range, the SE values increased, as a result in differences in participant behavior to changes in training direction, as noted previously^28^. Interestingly the generalization curve for the contextual movements (Fig. 2d) appears much wider and shallower than seen in the adaptation generalization curve (Fig. 2d). Moreover it also appears both shallower and wider than the contextual generalization curve seen in our previous study investigating passive contextual movements^28^.

#### Fitting the single field response

We fit the single field angular compensation data with a von Mises function, which is able to account for the circular nature of the response. As adopted in previous studies^24^,^28^, we make use of an offset as well as a width term (here *k*) and scaling factor as this is able to capture the underlying characteristics of the data across all experimental conditions. That is, the von Mises function can be used to represent that there is a directional tuning effect that exhibits a limited depth of modulation.

The adaptation tuning curve was fitted with a von Mises function (Equation 1) with a mean *μ* of 0.027 Rad (1.54°), *k* of 3.76 (equivalent σ of 29.5°), a scaling factor A of 3.78 and an offset B of 20.45 (Fig. 2c purple curve). The von Mises function provided a good linear regression fit to the mean experimental data with an R^2^ of 0.93, slope 0.93 and intercept 2.38%.

The contextual angle-tuning curve (Fig. 2d; blue curve) peaks around 0° with the same value and drops off slightly away from the training angle, but the tuning curve is quite wide and shallow and never drops below 72%. The contextual tuning curve was fitted with a von Mises function (orange curve) with a mean *μ* of −0.155 Rad (−8.90°), and *k* of 0.0072 (equivalent σ of 674°), a scaling factor A of 4735 and an offset B of −674. The von Mises function achieved a good linear regression fit to the mean experimental data with an R^2^ of 0.81, slope 0.81 and intercept 15.2%.

#### Kolmogorov–Smirnov statistic

To examine how the mean participant tuning curves compared to their fitted von Mises functions, the KS statistic was used. This was computed as the maximum absolute difference between the two respective normalized cumulative distributions tuning curves over a range of ±180°. The raw to fitted adaptation-tuning curve comparison yielded a KS value of 0.023 and the raw to contextual-tuning curve comparison yielded a KS value of 0.0049. In both cases, the KS values are well below the critical statistic (α = 0.05, n = 20) value of the test (0.294) indicating that the von Mises functions provide a good fit to mean participant data.

### Experiment 2: Generalization and interference of two motor memories

**Figure 3:**
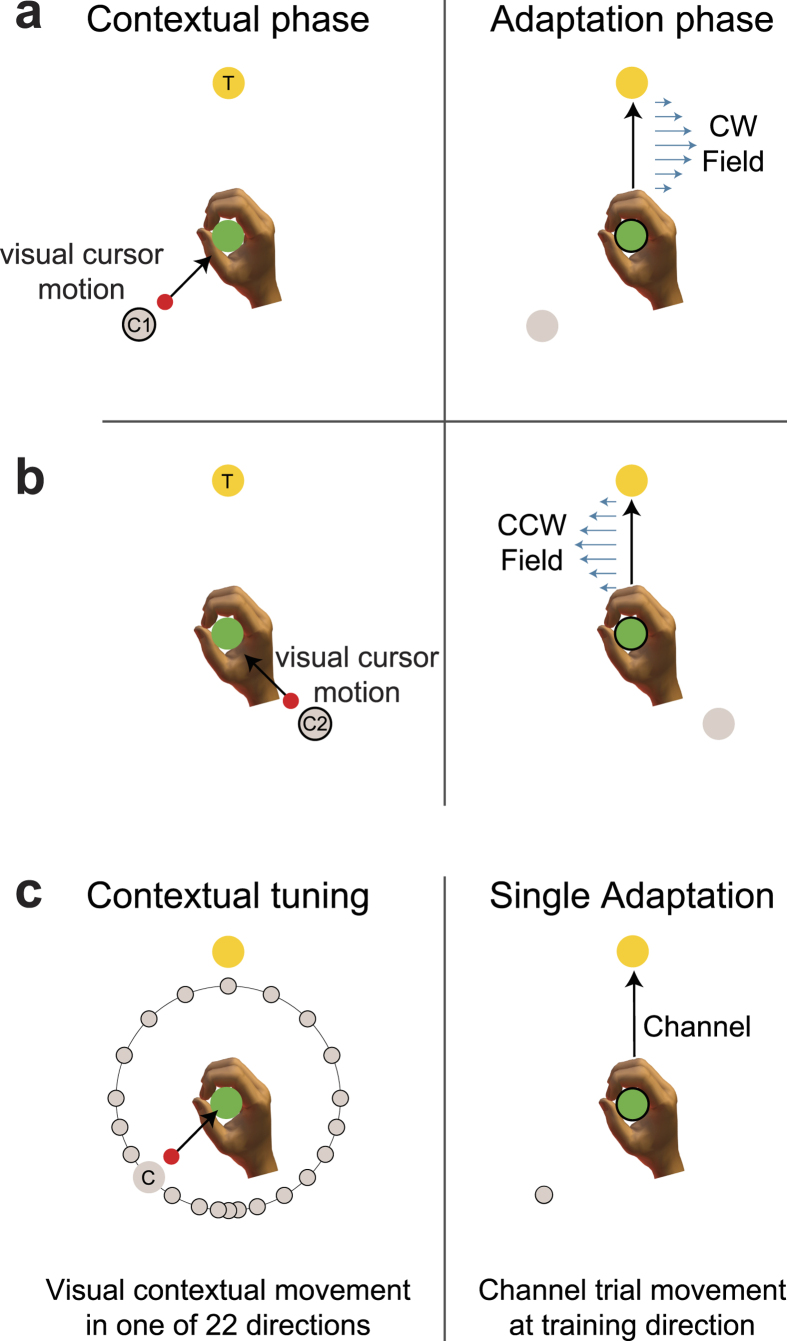
Interference tuning experimental paradigm. **(a,b**) Starting with the participant’s hand at the central location (green circle), the contextual phase consisted of visual observation of a cursor moving from one of two possible locations (C1 or C2, grey shaded circles, shown here for the ±45° condition) to the central location. Immediately after in the subsequent adaptation phase, the participant was required make an active movement to a final target location (yellow circle). During the latter movement they were subject to a curl force field of direction that depended on contextual movement starting location (here C1 relates to CW and C2 to CCW, although these were counterbalanced across subjects). (**c**) To examine contextual movement direction generalization, the visual cursor start position was varied and the adaptation movement was made to a single target in a mechanical channel to measure the predictive force.

**Figure 4:**
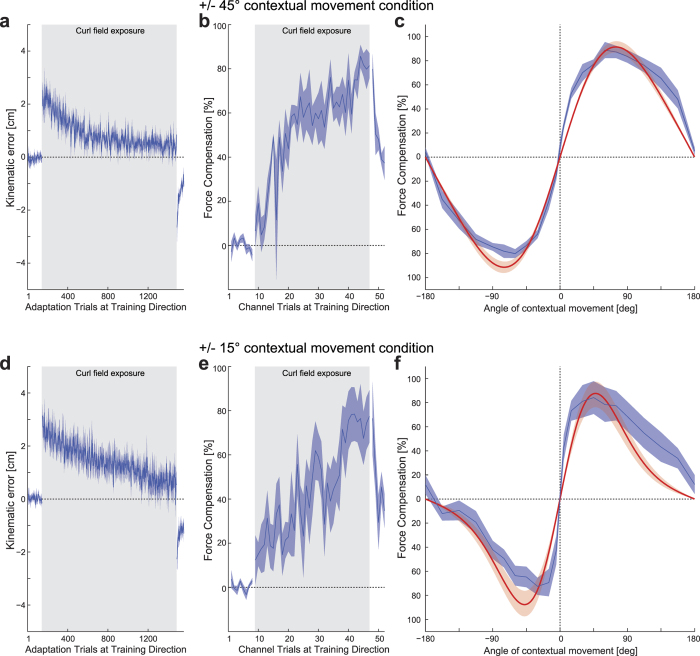
Interference tuning experimental results. (**a**) Mean kinematic error and SE per block across participants (solid line and shaded region respectively) for the adaptation movements using contextual movements at ±45°. The light blue shading indicates when the curl force field is applied. Although the two force fields produce error in the opposite directions, the sign of errors on trials on which the CCW field was presented have been reversed so that all errors in the direction of the force field are shown as positive. (**b**) Corresponding percentage mean and SE of predictive force compensation and (**c**) contextual movement tuning curve expressed as mean and SE percentage compensation (blue). The mean and s.d. of the best-fit von Mises functions are plotted in red. (**d–f**) As a-c but using contextual movements at ±15°.

The second experiment investigated the directional tuning of contextual visual motion using an interference task that involved simultaneously learning two opposing curl fields^26^,^28^. In this experiment the contextual effect of past visual cursor motion was essential to learn the appropriate compensation (Fig. 3). Two conditions were examined in separate groups of subjects, with visual contextual movements made either at ±45° or ±15° respectively. Each trial again consisted of a 2-part movement: an initial visual contextual movement followed by an active adaption movement. Each adaptation movement was associated with two possible visual cursor movement start locations (Fig. 3a,b left), which were predictive of the direction (CW or CCW) of the curl force field. In the ±45° condition, significant learning was observed during field exposure, as indicated by a large reduction in kinematic error (Fig. 4a) and a significant increase in predictive force compensation, with the latter reaching a value of over 80% (Fig. 4b). The ±15° group showed slower learning. This resulted in a slightly larger final level of kinematic error (F_1,1198_ = 25.8; p < 0.001) but with a final level of force compensation that was not significantly different from that of the ±45° group (F_1,154_ = 0.791; p = 0.375), see Fig. 4d,e. The dwell time between the prior visual contextual movements and the adaptation movements were 85.5 ± 14.6 ms and 95.8 ± 11.8 ms for the ±45° and the ±15° conditions respectively.

After participants had first learned to compensate both curl fields, generalization to changes in contextual movement directions was examined using channel trials in which the visual contextual motion starting point was varied, while the adaptation movement target location remained fixed to the 0° target (Fig. 3c, right). Tuning curves for the contextual movements plotted in terms of mean and SE of percentage compensation across subjects are shown in Fig. 4c,f (blue curve). A pronounced tuning response was found for both conditions, with compensation passing through zero around halfway between the contextual movement training directions. The transition became steeper as the two training directions moved closer together.

#### Fitting the interference responses

The mean participant data from the two tuning response conditions was fitted with von Mises difference functions (Equation 2). This formulation makes use of the same single *k* parameter for the two von Mises components, but with opposite signed means and scaling factors. The fitted responses are plotted in red on Fig. 4c,f. A good linear fit to the mean experimental results was found for the ±45° condition (R^2^: 0.97, slope 0.97, intercept 0.0%) and a reasonable fit was also found for the ±15° condition (R^2^: 0.89, slope 0.89, intercept 0.0%) groups. The ±45° group was fit with a von Mises with a mean *μ* of 43.8°, *k* of 0.286 (σ of 107°), and a scaling factor A of 46142. The ±15° group was fit with a von Mises with a mean *μ* of 14.7°, *k* of 1.25 (standard deviation σ of 51.3°), and a scaling factor A of 10975.

#### Predicting interference responses from single field responses

**Figure 5:**
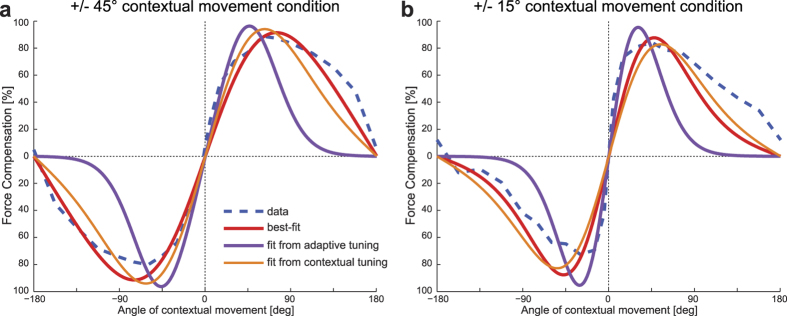
Interference responses can be predicted using tuning functions estimated from single a force-field condition. (**a**) Raw ±45° contextual movements tuning results are shown in dashed blue, and their von Mises fit is shown in red. The scaled predicted tuning functions made using the parameters estimated from the single field adaptation movements and contextual tuning are shown in purple and orange respectively. (**b**) Same as in (**a**) but for ±15° contextual movement condition.

We substituted the von Mises model *k* and scaling parameters estimated in Experiment 1 for the contextual or adaptation tuning conditions into the interference response (Equation 2), to examine if the characteristics of the single field response could explain the interference responses. To do so, we set the mean values in the interference to the training locations of the interference experiments (that to ±45° and ±15° respectively). The results of these predictions are compared with the raw mean participant responses (dashed blue curves) in Fig. 5.

Linear regression was used to examine the fit between the response estimated directly on the raw ±45° and ±15° interference data with the predictions made using parameters estimated in the single curl-field Experiment 1. A good fit was found between the ±45° raw interference data and the predicted single field contextual tuning response (R^2^ value of 0.98, achieved with a slope = 11.8) (Fig. 5a, orange line). A poorer fit was found between the raw interference data and the adaptation tuning response (R^2^ value of 0.71, achieved with a slope = 1.51) (Fig. 5a, purple line) suggesting that this tuning function based on adaptive movements does not predict the results of the interference experiment as well. However, for the ±15° interference study, both of the fits from the single field contextual experiments performed with a similar level. Specifically the linear regression fit was found between the ±15° raw data and the corresponding predicted single field contextual tuning response (R^2^ value of 0.76, achieved with a slope = 24.2). A similar fit was found between the raw interference data and the adaptation tuning response (R^2^ value of 0.76, achieved with a slope = 2.68).

We linearly scaled the predicted interference responses using coefficients obtained from the regression analysis and plotted them with the raw data and direct fit estimates. The ±45° results are shown in Fig. 5a. The raw experimental results are shown in dashed blue. The red curve shows the response functions estimated directly from the ±45° interference data, the purple curve shows the prediction made from the single force field adaptation data and the orange curve shows the prediction made from the single force field contextual data. It can be seen that there is good agreement between both scaled predicted curves based on the contextual tuning response and the raw data, but considerably less so for the scaled predicted curves based on the probe tuning response. Results for the ±15° interference data and the results are plotted in the same way in Fig. 5b. Although both of the predictive fits had equal R^2^ values for the fit to the raw data, the predictive fit based on the contextual movements with wide tuning (orange curve) matches much closer the direct fit (red curve) than does the one based on the adaptive movements (purple curve). This indicates that a simple von Mises difference model cannot capture the more non-linear boundary that arises in this condition. Close examination of the interference results show that although the difference between two von Mises tuning functions can model the general form of the interference response when the training angles are relatively far apart (i.e. ±45°), when the training directions are close together, they cannot account for the non-linearity present in the real data (i.e. ±15°).

Overall it can been seen that given suitable scaling, parameters estimated in a single force field condition could, to at least a first approximation, predict the directional tuning responses seen in the interference experiments. However it appears that when the training angles are close together, additional non-linear generalization behavior also plays a role in the motor memory recall process.

#### Comparison of generalization between visual and passive lead-in

**Figure 6:**
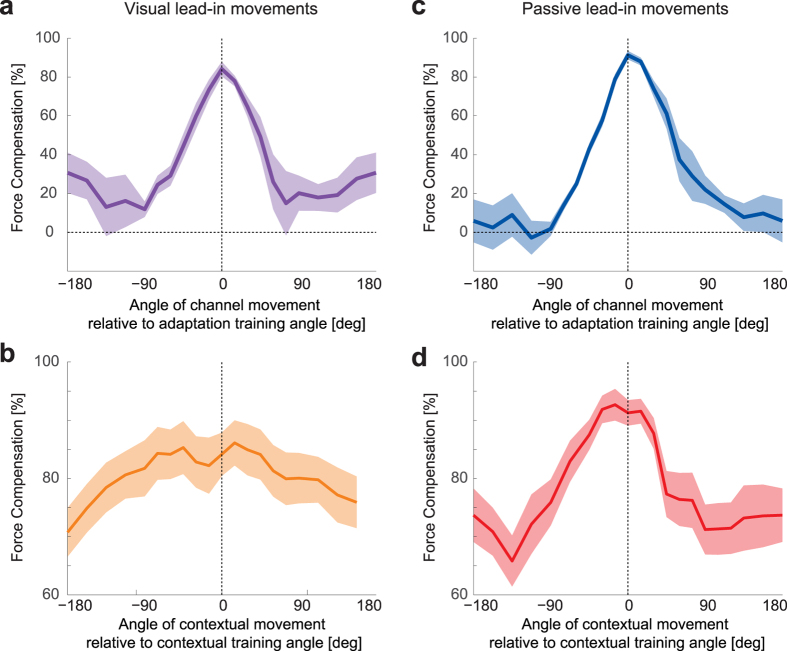
Comparison of experimental generalization functions for adaptive and contextual movements across the visual and passive lead-in conditions. (**a**) Tuning curve for the adaptation movement in the visual condition expressed as a percentage of perfect force compensation (mean ± SE across participants). (**b**) Corresponding tuning curve for the contextual movement in the visual condition. (**c**) Tuning curve for the adaptation movement in the passive condition determined from our previous study^28^. (**d**) Corresponding tuning curve for the contextual movement in the passive condition.

Our experimental design was similar to that of our previous study^28^ which examined passive contextual movements in order to facilitate comparisons across the results. Here we can directly compare the generalization data from the visual contextual movement condition (Fig. 6a,b) with those of the passive contextual movement condition (Fig. 6c,d) re-plotted from our previous results^28^. It can be seen that both the visual and passive generalization in the adaptive movements exhibit strong similarities (Fig. 6a,c). Both have a peak around 0°, fall towards zero compensation at around ±90°, have similar widths and exhibit an almost 100% modulation depth. In contrast, the visual and passive contextual tuning responses exhibit much shallower tuning curves, with modulation depths of only around 20% for the visual (Fig. 6b) and 30% for passive (Fig. 6d) conditions. One interesting feature to note is that the apparent width of generalization for the visual contextual movement (Fig. 6b) is much larger than that of the passive contextual movement (Fig. 6d). Instead the latter appears to be similar to that of the visual and passive probe tuning responses (Fig. 6a,c).

**Figure 7:**
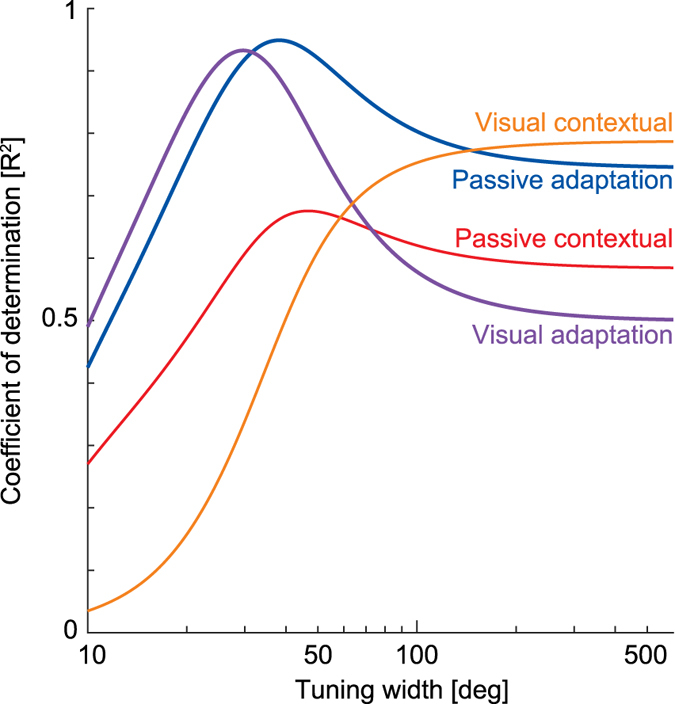
Sensitivity of single force field tuning functions to von Mises tuning width. The effect of Mises tuning width (displayed as σ) on the R^2^ match between model predictions and the mean experimental data is plotted over a range of widths between 10°–600°. Each curve presents the R^2^ match to the four single force field condition responses. That is for the adaptation movement generalization (dark blue) and the contextual movement generalization (red) for the passive lead-in, as well as the adaptation movement generalization (purple) and the contextual movement generalization (orange) for the visual lead-in.

It could be argued that the tuning functions, particularly those of the interference experiment, might not be sensitive to the variations in the best-fit width parameters in the current and previous study. Here we investigate to what degree the fit to the tuning functions depends on the tuning widths. To examine the effect of the tuning width parameter on the behavior of the von Mises function used to model single curl field generalization, we calculated the R^2^ between the mean experimental responses across participants to the von Mises model predictions for a range of tuning widths, represented as the parameter σ. We calculate results for both the adaptation tuning function and the contextual tuning function from the current single field visual data results, as well as those from our previous passive data^28^ (Fig. 7).

It can be seen that as the value of σ changes, it has a substantial effect on the R^2^. In particular as the value of σ increases, the R^2^ value is initially quite sensitive to σ until a peak is reached, after which it’s sensitively to σ reduces. As expected, each experiment condition has different optimal σ values that maximize R^2^. For the visual lead-in adaptation generalization and context condition, R^2^ peaks at σ = 29.9° and σ = 1000° (highest last value tested) respectively. These σ values yield R^2^ measures that are in good agreement with those discovered by direct fits to the experimental data, which found values of σ = 29.5° and σ = 674° respectively. The small discrepancies in parameter values that arose can be explained by the differences in the two fitting procedures and that in the former case, the von Mises mean parameter was set to zero and not estimated from the data. For the passive lead-in adaptation movement generalization and contextual movement generalization, R^2^ peaks at σ = 38.5° and σ = 46.4° respectively. If we directly refit our previous data using the von Mises functions, we obtain width parameter values of σ = 38.5° & σ = 41.2° respectively. It can be seen that all of these σ values yield R^2^ measures close the peaks values seen in the R^2^ plots. What is clear from these results is that the visual contextual movement tuning exhibits a much broader width than that of the adaptive movements or our previous passive contextual movements. However the exact width of this function is less clear as all values between 90° and 1000° provide a good fit to the data.

**Figure 8:**
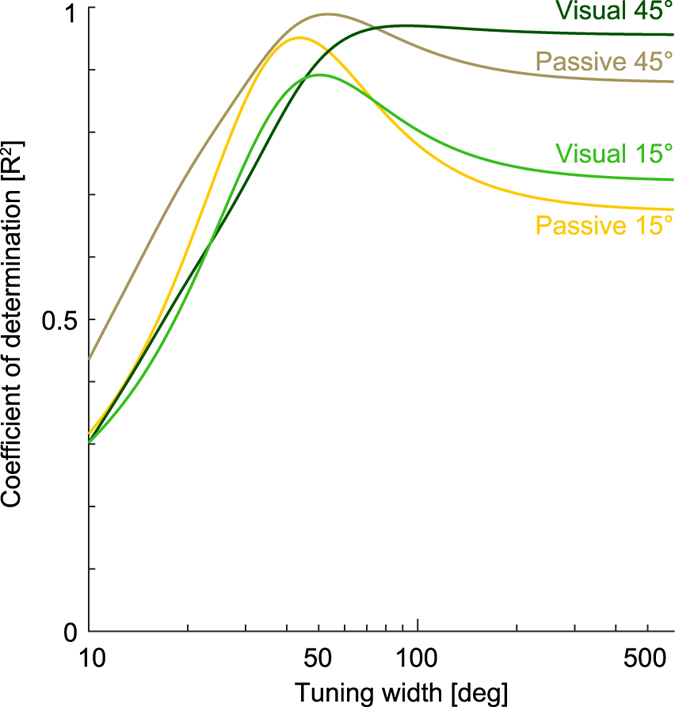
Sensitivity of interefence tuning functions to von Mises tuning width. The effect of Mises tuning width (displayed as σ) on the R^2^ match between model prediction and interference datasets are plotted over a range of widths between 10°–600°. Each curves represent the R^2^ matches to the four interferences condition responses: namely to the ±15° (light green) and ±45° (dark green) visual conditions and to the ±15° (yellow) and ±45° (brown) passive conditions.

In a similar fashion we also examined the sensitivity of the interference responses to the width parameters (Fig. 8) for both the ±15° and ±45° visual and passive conditions. It can be seen that the tuning parameter σ substantially affects the interference characteristics and therefore R^2^. Again these curves rise rapidly to a peak and then slowly fall off as σ increases further. That is, the R^2^ value is initially quite sensitive to σ until a peak value R^2^ is reached, after which it’s sensitively to further changes in σ reduces significantly. For the ±15° and ±45° visual lead-in condition, the R^2^ peaks at 50.4° and 92.2° respectively, whereas direct fitting on the experimental data found σ values of 51.3° and 107° respectively. For the ±15° and ±45° passive lead-in condition of our previous paper^28^, the R^2^ peaks at 43.9° and 53.3° respectively. Direct fits to the experimental data found σ values of 44.9° and 64.6° respectively. Once again all of these σ values yield R^2^ measures close the peaks values seen in the R^2^ plots. It can be seen that any von Mises width value above 80° provides an equally good fit to the visual ±45° lead-in condition. This matches reasonably well with the visual contextual tuning single field results (Fig. 7) suggesting that the visual contextual movements exhibit a broader tuning than those of passive movements. The results of the visual ±15° lead-in interference study demonstrate that, when required, the tuning can be narrower, but it is important to recall that none of the simple additive von Mises functions captures the shape of the experimental results in this condition.

## Discussion

We investigated if the contextual effect of immediate past visual contextual movement exhibits an angular dependency on direction. After adaption to a single curl force field, the tuning curve for the adaptation movement peaked at 0° (trained direction) and exhibited generalization of predictive learning away from the trained movement direction which decreased smoothly until no predictive forces were present at ±90°, consistent with previous studies^9^,^10^,^40^. In contrast, the tuning function for the visual contextual movements was found to be wider and shallower. To shed some light on these phenomena, we adopted a computational modelling approach to look for consistencies across the two experimental conditions.

We found that the single field contextual and adaptation tuning responses could be well fitted with von Mises functions. Moreover, the von Mises distribution parameter *k* estimated from the contextual movements in the single field experiment could predict the response curves seen in the interference experiments. In particular, the wide tuning curve in the single field condition was consistent with the results of the interference experiments, in which the responses in both ±45° and ±15°conditions could be predicted from the broad visual contextual tuning response. The compensation response against movement angle in the ±45° condition showed an almost sinusoidal response. This is expected because as the tuning width increases, the difference between two von Mises functions becomes sinusoidal. In contrast, the ±15° condition exhibits a steeper transition between the two training directions. The phenomenon of an increasingly non-linear transition as training directions become closer was also seen in our previous passive movement tuning study^28^.

On the basis of these two sets of experiments we suggest that even though past visual movements only exhibits a slight effect of motor learning in subsequent movements during the learning of a single force field, this contextual effect can be enhanced dramatically during an interference task when it can assist with the task. This phenomenon was also seen in our previous passive movement context study^28^. This suggests that in task-relevant situations, a relatively weak contextual effect arising from past visual movements can be given sufficient weighting to enable it to play a strong role in motor learning and recall.

Two studies have recently characterized the angular tuning responses for past contextual movements using either passive^28^ or combined active and visual movements^24^. In both studies, a pronounced tuning response was observed for contextual movement direction, which was subsequently modeled with Gaussian functions. Although the paradigms are slightly different to the one used in our current study, these results can be compared with our current visual motion study. Here we have used the von Mises function to model the tuning responses as it is more appropriate than the previously used Gaussians, to model circular responses. The von Mises parameter *k* is also related to the Gaussian parameter σ (1/*k* is analogous to σ^2^) thereby making comparisons straightforward.

The generalization function of the adaptation movement has been examined multiple times using a variety of experimental designs. In our current study we found a width of the tuning function of m σ = 29.5°, whereas our previous study^28^ estimated widths of 39.97°. Looking at learning in multiple directions and estimating the adaptive tuning functions from changes in the errors from direction to the next, the half-height value was estimated to be about 40° 10 which corresponds to a σ = 34°, with similar results in a comparable study^9^. Therefore these studies, using a variety of techniques, have all estimated similar widths of the tuning functions. More relevant for the investigation of contextual effects, are the estimated widths of the tuning functions for the contextual movement. In our current study we found that the visual contextual movement had an equivalent σ = 674°. This generalization function was also shallow, with a fall in average compensation across participants from 85% at the training direction to 72% at the minimum (a difference of only 13%). This tuning width is much broader than previously found for either passive movements^28^ (σ = 42.76°) or active movements^24^ (σ = 27.4°). Moreover both of these previous functions exhibited a larger drop away from the peak adaptation magnitude with reductions of 25% and 46% respectively, although the differences - compared to the current study - could be related to the large width of the tuning function found for visual motion. A comparison of these results suggests that within a single force field learning task, active movements have a stronger contextual effect than passive motion, which again has a stronger effect than visual motion.

In our second experiment, generalization was examined in an interference task. This involved participants learning two opposing force fields in a paradigm in which each force field direction was associated with a distinct visual contextual movement. When the generalization function was fit with two von Mises functions, this led to tuning widths for the visual contextual movements with equivalent σ = 107° and σ = 53° for the ±45° and ±15° conditions respectively. These are much broader than those found previously^28^ for passive contextual movements where fitting the experimental data yielded σ = 43.32° for both for the ±45° and ±15° conditions. Similar response widths were also found for active movements^24^ at the ±45° directions where the widths varied between 39–57°. Again, it can be seen the passive and active contextual tuning width are comparable, whereas the visual tuning width is larger. However the best-fit tuning widths were still smaller than those found in the single field condition. It is important to note that we do not claim that the underlying neural tuning is specifically von Mises or Gaussian in nature, but rather that this model is a simplified fit to the data that predicts response in the interference experiment with minimal parameters. It can be seen that the widths of the von Mises functions, used to model the underlying neural tuning functions, are well preserved across many different studies examining generalization and interference^9^,^10^,^24^,^28^. Moreover, the larger widths of the tuning functions found for visual contextual movements better explains the results of the visual interference study than the narrow tuning of the passive or active movements. However, it is important to note that none of the simple additive von Mises functions captured the full shape of the experimental results in the 15° visual interference condition. This may indicate additional non-linear generalization behavior or could suggest that the generalization may arise through a combination of neural basis functions with many tuning widths. That is to say, while visual contextual movements may naturally exhibit broad tuning, the sensorimotor control system may be able to utilize narrower tuning functions when required for the task.

Both here and in our previous study^28^, we suggested that the tuning parameters estimated in the single force field can predict the interference responses. Although Sarwary and colleagues^24^ state that the single field conditions do not predict the interference results, they do not directly fit their single force field tuning parameters to the interference tuning data. Instead they simply compare the parameters after independently fitting the results from the two experiments. However, as we demonstrate in both of our studies, the width of the tuning function obtained from the static field case leads to a good prediction of interference response despite differences in the width parameters from the best-fit functions. Specifically, even though the best-fit single force field tuning width (σ = 674°) was much larger than either the best-fit widths from the ±45° (σ = 107°) and ±15° (σ = 53°) conditions, a scaled version using this parameter was still able to fit the interference response curve with both R^2^ > 0.75.

We found that the final compensation levels reached after learning with the visual context were similar between both group ±45° and ±15° groups. This was not the case for our passive context interference experiments^28^. In addition, the visual context interference tuning curves peaked at higher values than those in the respective passive condition. These results from the visual contextual motion experiment may appear surprising, as one might expect that as the tuning widths become wider, we would expect larger overlap between the two training conditions and therefore more interference between the learning of the two force fields. However the results of fitting the broad contextual tuning functions from the single force field to the interference experiments demonstrates that broad tuning functions can still produce such high adaptation curves provided the gain of the individual functions is sufficiently high (Fig. 5).

There have been several proposed mechanisms to account for contextual recall effects in motor learning^41^,^42^,^43^,^44^, which often rely on a single contextual or responsibility signal which gates the specific controller used for each task. Lee and Schweighofer^44^ proposed that this contextual signal is binary, specifying a specific slow learning process out of many. In our current task however, we would suggest that there is no longer a binary signal that gates each controller but rather that the output is a weighted combination of multiple controllers or processes. This allows the sensorimotor system to makes predictive estimates of the output force for contextual movements between the two learned training directions, produced by an addition of the output of two tuning functions. In our case the prior sensory states of the arm or cursor are used as this contextual signal. We suggest that the reason that visual, passive and active movements can all act as contextual cues is because they are all combined to produce an estimated state of the limb. This might suggest that state estimation, suggested to occur within the cerebellum^45^ might be one of the critical signals for these contextual effects. Previous work has indeed shown that changes of state arising from changes in physical location^21^,^46^ or even simply visual location^16^,^20^ exhibit strong contextual effects and a reduction in interference. Although the exact neural implementation of these contextual effects is still unknown, we have theorized about possible neural mechanisms in our previous papers^26^,^27^,^28^.

Our results show that visual contextual effect of past movement influences predictive force compensation during arm movement tasks. We found that visual contextual tuning was much broader than found previously for active and passive contextual movements. Such a wide tuning response suggests less angular sensitivity to moving visual objects than those invoked from actual movements of the hand. Consequently one might expect that visual contextual movement would generate a less differentiated response than that produced by active past movement. Overall, a comparison of the results in from the current study with previous work on passive and active past contextual movements indicates that the modality of past movement plays a strong role in the characteristics and strength of its contextual effects.

## Methods

A total of 20 participants (9 male, 11 female; age = 21.7 ± 2.83 mean ± sd years) were randomly allocated to two experiments. Participants were naïve to the aims of the experiments and provided written informed consent. The University of Plymouth Faculty of Science and Technology Human Ethics Committee approved the protocol. The methods were carried out in accordance with the approved guidelines. All participants were right handed based on the Edinburgh handedness questionnaire^47^. We note that methods used here adopt a similar paradigm to one used in our previous study that examined the generalization of passive past movement^28^.

### Apparatus

Experiments were performed using a vBOT planar robotic manipulandum and associated virtual reality system^48^. The vBOT is a custom built back-drivable planar robotic manipulandum, which exhibits low mass at its handle. Handle position is measured using optical encoders sampled at 1000 Hz, and torque motors allow end-point forces to be specified. The position signal is used unfiltered, whereas to reduce the effects of noise, the velocity is computed by fitting a quadratic equation of motion, assuming constant acceleration, over a window that consisted of the 30 most recent position samples and associated time stamps. The vBOT is equipped with a force transducer (Nano 25; ATI) mounted at the handle to measure the applied forces. Prior to digitization, the output channels of the force transducers are low-pass filtered at 500 Hz using analogue 4th pole Bessel filters. Participants were seated in a sturdy chair in front of the apparatus and firmly strapped against the backrest with a four-point seatbelt to reduce body movement. Participants held the robot handle in their right hand while an air sled supported their right forearm on an air table, thereby constraining movement to the horizontal plane. Visual feedback was provided using a computer monitor mounted above the vBOT where the scene was projected veridically to the participant via a mirror, such that the visual cursor appeared to the participant in the same plane and at the same location as their hand. Participants were prevented from viewing their hand directly, and instead the virtual reality system was used to overlay images of the starting location, via point, final target, (all 1.25 cm radius disks) and a hand cursor (0.5 cm radius red disk) in the plane of movement. In all experiments, data was collected from the manipulandum’s encoders and force transducer at 1000 Hz and logged to disk for offline analysis using Matlab (Matlab, The MathWorks Inc., Natick, MA, USA).

### Force Fields

Participants performed reaching movements in which either a null field or a velocity-dependent viscous curl force field^4^ was presented. In the curl force field condition, the force experienced at the manipulandum’s handle was given by:


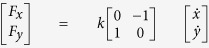


where *k* was set equal to ±13 N m^−1^ s. The sign of *k* determines the direction of the force-field (CW or CCW). In Experiment 1, each participant only experienced a single force field direction. In Experiment 2, each participant experienced both force field directions where the specific direction depended on the contextual phase of the trial.

In all experiments, the relationship between visual contextual movements and curl field direction (CW/CCW) was counterbalanced across participants to minimize any effect arising from the interaction of movement direction and curl field direction. The force-field adaptation was estimated by measuring kinematic error on field trials and force compensation on channel trials.

### Protocol

On each trial, the starting location for the visual contextual movement, the central location and final target and the cursor were displayed throughout the entire task. The vBOT then moved the participant’s hand (from its location in the workspace arising from the previous trial or initial starting location) to the central location (following a minimum jerk trajectory). Once the handle remained within the central location at a speed below 0.1 cm/s for 500 ms, participants were cued by an acoustic tone indicating the start of the trial. The first part of the trial was a purely visual contextual movement of the cursor that followed a minimum jerk trajectory of duration 640 ms from the start to the central location. Once the visual cursor reached the central location, participants immediately performed an active movement to the final target position. Participants were encouraged to perform the second movement immediately after the visual contextual movement reached the central location, and were required to do so within 250 ms or the trial was aborted (and then repeated). In practice participants generally remained at the central location for a short time, and the dwell time period for which they remained within the radius of the central location disc with a speed less than 5 cm/s was calculated. Moving towards the final target before the cursor reached the central location also resulted in an error and the trial was also aborted and repeated.

This second movement constituted the adaptation movement, as it was only during this movement that force fields were introduced. If the duration of the adaptation movement (measured from the time the cursor had moved from the central location until it entered the target location) was between 150 and 300 ms, a “correct speed” message was displayed; otherwise the appropriate “too fast” or “too slow” warning was shown. Throughout each experiment participants were provided with a short rest break approximately every 200 trials (195–205 trials), although they could also take a break at any time by releasing the handle switch of the manipulandum.

### Experiment 1: Generalization of a single motor memory (n = 8)

The first experiment examined the directional tuning of both contextual visual motion and the active adaptive movements using a single curl field-learning task. In this experiment the past visual motion context is not required in order to learn the compensation (Fig. 1). Each trial consisted of a 2-part movement. Initially the participants’ hand was pulled to the central location by the robot. If we define 0° as the direction straight ahead, then the starting position for the visual cursor movement was located at 180° (Fig. 1a). Participants then experienced a visual 10 cm contextual movement of the cursor to the central location. Immediately afterwards the participants were required to make a 12 cm active adaptation movement from this central location to a final target located straight ahead at 0°. Prior results have demonstrated that fixation on the central via location or free choice on eye fixation did not change the underlying contextual effect of visual movement^26^. We therefore only instructed the participants to move to the final target position when the cursor reached the central via position and did not constrain eye gaze location. This active movement could be performed in a null field, a mechanical channel, or a curl force field (Fig. 1a right).

Throughout the experiment, to assess feedforward compensation to the curl force field, channel trials^49^,^50^ were employed in which the contextual and adaptation movement directions were the same as during field training. In such a channel trial, the adaptation movement was confined to a simulated mechanical channel with a spring constant of 10,000 N/m. The channel was only present during the second adaptation movement.

To examine the generalization effect of contextual movement direction, the final target location remained fixed at the 0° target and the location of the visual contextual motion starting point was varied. The latter was selected pseudo-randomly from 20 starting points, concentrated around the contextual training direction, but spanning the full possible 360° range (Fig. 1b). Specifically the contextual movement deviated from training angle by 15° for the ±90° range, and then by 30° for the remaining full ±180° range. Again, a channel was applied during the adaptation part of the movement. The contextual movement generalization data was fitted using a parametric tuning curve.

To examine the generalization effect of adaptation movement direction, the visual contextual motion training direction remained fixed at the training location while the target locations varied. The latter were selected pseudo-randomly from 20 target locations concentrated around the adaptation movement training direction, again spanning the full possible range of 360° (Fig. 1c). The adaptation movement direction deviated from the training angle by 15° for the range ±90°, and then by 30° for the remaining full ±180° range. All adaptation movements in the 20 directions were performed in a channel. The adaptation movement generalization data was similarly fitted using a parametric tuning curve.

Experiment 1 was run in six distinct phases and consisted of 1970 trials arranged as follows:

Pre-exposure: This was carried out to estimate baseline movement performance and enable participants to practice the task. This phase consisted of 6 blocks of 10 null trial blocks in which no forces were applied. A block consisted of 9 Null trials and 1 channel trial (Total 60 trials: 54 null trials, 6 training direction channel trials).

Pre-exposure Generalization Testing: This was used to ensure participants had prior experience of the wide range of generalization direction trials that would take place during the generalization phase of the experiment. This phase consisted of 2 sets of blocks containing 40 field and 40 tuning channel trials. In each block, every generalization condition was experienced pseudo-randomly within a channel. In total, there were 2 repetitions of each of the 40 channel trials. (Total 160 trials: 80 field trials, 80 generalization direction channel trials).

Pre-exposure: The null pre-exposure phase was repeated again before commencing the training. This consisted of 6 blocks of 10 trials in the null force field (Total 60 trials: 54 null trials, 6 training direction channel trials).

Exposure: During the exposure phase, the novel dynamics (curl force fields) were introduced. The phase consisted of 35 blocks of 10 field trial blocks (Total 350 trials: 315 field trials, 35 training direction channel trials).

Exposure Generalization Testing: Generalization of the learned predictive compensation was investigated by pseudo-randomly interspersing channel trials for a range of contextual and adaptation movement angles with the field exposure trials. This consisted of 10 sets of 128 trial blocks, which meant that there were 10 repetitions of each of the 40 channel trials. (Total 1280 trials: 880 field trials, 400 tuning direction channel trials).

Post-exposure: This was used to examine movement trajectories after the dynamic force fields were removed. This consisted of 6 blocks of 10 trial null trial blocks, in which no forces were applied. A block consisted of 9 Null trials and 1 channel trial (Total 60 trials: 54 null trials, 6 training direction channel trials).

Experiment 2: Generalization and interference of two motor memories (n = 12)

The second experiment investigated the directional tuning of the contextual effect of visual contextual movement, using an interference task^26^ (Fig. 3). Each trial again consisted of a 2-part movement, ending at a target located at either 0° or 270°. For both groups, the participants’ hand was first pulled to the center location. For each of the adaptation movements, there was one of two possible start locations for the visual cursor movement, located at either side of the middle line towards the central via point (Fig. 3a,b left). One group of participants (n = 6) experienced visual contextual movements at ±45° relative to the direction of each of the adaptation movements, whereas a second group of participants (n = 6) experienced visual contextual movements at ±15°. Immediately after participants experienced the 10 cm visual cursor motion to the central location, they were required to make an active movement from this central location to the final target location (Fig. 3a,b right). The first visually observed cursor motion could act as a source of contextual information for the second active movement, during which a randomly selected clockwise or counter-clockwise velocity-dependent curl field was applied. The direction of the curl field was associated with the starting location of the contextual movement.

To assess learning of the two opposing curl-fields throughout the entire experiment, channel trials were included for the active (adaptation) movements to the 0° target. One trial was preceded with the visual contextual movement associated with a CW field and the other trial was preceded with the visual contextual movement associated with a CCW field (randomizing which came first within each block). As in Experiment 1, channel trials were also used to examine the generalization of learning as the visual contextual motion starting point was varied, while the adaptation movement target location remained fixed to the 0° target (Fig. 3c, right). The 22 possible starting points were concentrated around the midpoint between the two contextual training directions and spanned the full 360° range. Specifically visual contextual movements started from points located at 180°, deviating by 5° for the first 2 data points, for 15° for the ±90° range, and then by 30° for the remaining full ±180° range (Fig. 3c, left). The particular channel trial used on any given movement was selected pseudo-randomly.

The organization of trials in each instance of Experiment 2 was similar to the procedure adopted in Experiment 1. There were 1904 trials overall, organized as follows:

Pre-exposure: This consisted of 2 blocks of 40 null trial blocks, in which no forces were applied. A block consisted of 36 Null trials and 4 channel trials (Total 80 trials: 72 null trials, 8 training direction channel trials).

Pre-exposure generalization testing: This consisted of a block of 22 field trials and 22 tuning channel trials, in which all generalization conditions were experience pseudo-randomly within a channel (Total 44 trials: 22 field trials, 22 generalization direction channel trials).

Pre-exposure: This consisted of 2 blocks of 40 trials in the null field (Total 80 trials: 72 null trials, 8 training direction channel trials).

Exposure: This consisted of 15 blocks of 40 trials (Total 600 trials: 540 field trials, 60 training direction channel trials).

Exposure generalization testing: This consisted of 10 blocks of 102 trials of which 22 were generalization channel trials. (Total 1020 trials: 800 field trials, 220 tuning direction channel trials).

Post-exposure: This consisted of 2 sets of 40 trial null trial blocks, in which no forces were applied. A block consisted of 36 Null trials and 4 channel trials (Total 80 trials: 72 null trials, 8 training direction channel trials).

### Analysis

The data was analyzed offline using Matlab. To examine learning and generalization two measures were used; kinematic error on the adaptation movements in which either the null field or curl force field trials were present and force compensation on the channel trials.

### Kinematic error

The kinematic error was calculated on each adaptation movement as the maximum perpendicular error (MPE) of the hand path relative to a straight line joining the start of the movement to the center of the target location. For each participant, the MPE for all field exposure trials was averaged over 4 trials, with the sign flipped appropriately so that errors from CW and CCW field trials could be appropriately combined. The mean and standard error (SE) of MPE was then computed across all participants.

### Force compensation

In order to look for evidence of feed-forward adaptation, as opposed to relying on a reduction in kinematic error during force field learning which can also arise from muscle co-contraction induced limb stiffness^51^,^52^,^53^, channel trials were randomly interspersed in each block of field exposure trials. Using this technique, measurement of endpoint forces at the handle onto the channel wall can estimate the amount of specific adaptation learned during field exposure^54^. More specifically, the force produced by participants perpendicularly into the wall of the simulated channel was first integrated across the adaptation phase movement. This integrated force was then divided by the value required to completely compensate for the field, as determined by the field strength and the corresponding integrated movement velocity on each trial. This yielded an estimate of the level of force compensation present as field exposure progressed^26^,^27^. The mean and SE force compensation was computed for all channel trials within each block across all the participants.

### Statistics

To compare the levels of adaptation for the two interference studies (±45° and ±15° groups) two ANOVAs using the generalized linear model in SPSS were run on the MPE and force compensation data. For each ANOVA, the values over the last third of the exposure phase were compared with a factor of experimental group (2 levels: ±45° and ±15°). Significant differences were determined at the 0.05 level.

### Modeling tuning responses with von Mises functions

In previous passive and active tuning contextual movement studies a Gaussian function was used to model the full 360° angular tuning response^24^,^28^. In these cases this approach was acceptable because of the narrow nature of the tuning responses involved. That is, with σ of around 40°, the response will have essentially fallen to zero at 180° from the training direction, so to a first approximation, boundary effects can be neglected. However, in the current study, contextual movements exhibit a wider tuning response. Therefore the interference responses cannot simply be modeled as the difference between two Gaussian functions, since in this case the tuning curves will have significant responses at an angular deviation of 180° away from the training direction which would overlap. To resolve this issue, here we use the circular von Mises function to model tuning functions and to predict interference responses. Note that we do not claim that the underlying neural tuning is a von Mises function, but rather that this model is a simplified fit to the data that can predict responses in both the single force-field and the interference experiment with minimal parameters.

### Modeling tuning response with a single von Mises function

In the single curl field Experiment 1, the generalization channel trials correspond to both contextual and adaptation movement angles. The angular deviations from the training angle were first used to plot these two tuning functions. The sign of the counterbalanced dataset results were appropriately changed so that all participants’ data in a given experiment could be combined into a single average tuning curve. The angular tuning curves for both contextual and adaptation movement constituted circular data that each had a single peak at the training direction, and a response that fell off either side from this direction. We fitted the Experiment 1 tuning response curves using a single von Mises model function 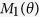:


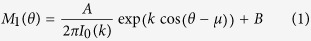


where *θ* is the deviation angle from the training direction in degrees, *μ* and *k* are the mean and width parameters of the von Mises function respectively and 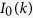 is a Bessel function of order zero. A is a scaling factor and B is an offset. We note that 1/*k* is analogous to the Gaussian distribution variance σ^2^.

### Modeling interference response as the difference of two von Mises functions

In the interference Experiment 2, the generalization channel trials were carried out in two contextual movement angle conditions (namely either ±45° or ±15°). In each case the tuning curves were bimodal, with peak magnitudes of opposite sign occurring near the training directions and passing through zero around the 0° movement directly between them. We modeled this response as the difference of two von Mises functions having the same width parameter *k* but opposite means and scaling factors, using the von Mises difference model function


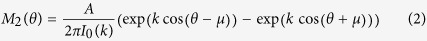


where *θ* is the deviation angle from the central direction in degrees, and *μ* and *k* are the mean and with parameter of the von Mises function respectively, 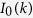 is a Bessel function of order zero and A is a scaling factor. We note that the offset factor B needed to model the single curl-field response cancels out in this equation.

### Fitting mean response data

The average compensation data across participants was fitted using non-linear optimization (with the Matlab function fmincon) that minimized the root mean square error between the appropriate von Mises model (Equations 1 or 2) to the averaged participant data. That is, the object function minimized the error E which is the Euclidian distance between the model and response curves, as given by:


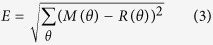


where *θ* is the movement angle, R(*θ*) is the mean participant response at angle *θ*, and M(*θ*) is the model response at angle *θ* (using either Equation 1 for 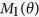 or Equation 2 for 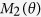 in Experiments 1 or 2 respectively). To ensure a good fit was achieved on the single dataset in Experiment 1 and both datasets in Experiment 2, for each condition the optimizations were run ten times from pseudo-random starting positions and the fit with the lowest error used.

### Estimation of fitted response confidence limits

Bootstrap statistical analysis was performed to provide confidence intervals for the fitted response parameters. This was achieved by first randomly choosing participants with replacement, computing the average tuning curve over the chosen participants and then performing the parameter fitting. In Experiment 1 this involved the random selection of 8 participants, and in Experiment 2 the random selection of 6 participants. This procedure was repeated 1000 times and the results were used to estimate the mean and standard deviation of the fit.

## Additional Information

**How to cite this article**: Howard, I. S. and Franklin, D. W. Adaptive tuning functions arise from visual observation of past movement. *Sci. Rep*. **6**, 28416; doi: 10.1038/srep28416 (2016).
